# ‘A sad inheritance of misery’: the cultural life of hereditary scrofula in eighteenth-century England

**DOI:** 10.1017/mdh.2023.37

**Published:** 2024-01

**Authors:** Noelle Dückmann Gallagher

**Affiliations:** The University of Manchester, Manchester, United Kingdom of Great Britain and Northern Ireland

**Keywords:** England 1660–1800, scrofula, disease, heredity, literature, art, print culture

## Abstract

This essay argues that scrofula was one of several disorders, including gout, rickets, and venereal disease, that were ‘rebranded’ as hereditary in response to broader cultural changes that took place during the Restoration and eighteenth century in England. While the purposes of scrofula’s recategorisation were more political than medical, they resulted in this heretofore relatively obscure childhood ailment assuming a new prominence within both medical and popular discourses of the period. Scrofula became both emblem and proof of the links between sexual promiscuity, financial profligacy, and physiological degeneration, its symbolic status reinforced by the legal and moral language used to model processes of hereditary transmission. By likening the inheritance of scrofula to the inheritance of original sin—or, more commonly, to the inheritance of a ‘docked entail’ or damaged estate—eighteenth-century writers and artists not only made this non-inherited ailment into a sign of catastrophic hereditary decline; they also paved the way for scrofula to be identified as a disease of aristocratic vice, even though its association with crowded, unsanitary living conditions likely made it more common among the poor. By the same token, financial models of disease inheritance facilitated a bias toward paternal transmission, with scrofula often portrayed as passing, like a title or an estate, from father to son rather than from mother to daughter.

## Introduction

In his 1676 *Treatise of the Kings-Evill*, royal surgeon Richard Wiseman explained that the ultimate causes of scrofula—an infection of the cervical lymph nodes also known as struma or ‘the king’s evil’— were ‘somewhat hard to be enumerated’, since they could include ‘Air, Diet, Exercise, natural Complexion, hereditary Affections’, and other factors.^1^ However, within a few decades of Wiseman’s publication, medical and popular discussions had begun to highlight one particularly pernicious cause: while the disorder *could* occur adventitiously, most often it was inherited, passed down by irresponsible parents to their innocent, unwitting offspring. So broad and persistent was this shift in opinion that practitioners like Thomas White, surgeon to the London Dispensary, were still attempting to refute the ‘common idea that Struma is an hereditary disease’ some 100 years after Wiseman’s publication.^2^ In his own *Treatise on the Struma or Scrofula* (1784), White confessed that he was unsure ‘at what precise period the opinion arose, that the Scrofula differed so peculiarly from the generality of diseases by the circumstance of hereditary transmission’, but he was convinced by his own clinical observations that this view was ‘very erroneous’.^3^

As it happens, White was correct—both in his suspicion that views of scrofula had changed at a particular time and in his conviction that scrofula was *not*, in fact, hereditary. Scrofula—or mycobacterial cervical lymphadenitis, as it is known today—is caused by mycobacteria, the same bacteria that can, in their tubercular form, lead to pulmonary tuberculosis (though early modern physicians would not have known of this link).^4^ It arises when these mycobacteria invade the lymph nodes of the cervical spine, and, if left to progress, it can lead to unsightly ulcers on the patient’s neck and face. It is not genetically inherited, nor is it very contagious, particularly in the non-tubercular form in which it often appears in children.^5^ Today, scrofula is almost unheard-of in developed nations, though it occasionally appears in immunocompromised patients or among those living in crowded, unsanitary conditions. It is now treated with antibiotics, but even in a pre-antibiotic age, it is likely to have sometimes resolved without treatment.^6^

Given that bacteria can mutate over time, it is entirely possible that a higher proportion of childhood scrofula cases in eighteenth-century Britain were of the more serious, tubercular sort; this could mean that the disease was potentially more infectious within households as well as more dangerous to sufferers. It is also likely that some—perhaps many—of the conditions diagnosed as scrofula in the eighteenth century would not correspond with the mycobacterial cervical lymphadenitis of today. Yet even keeping these allowances in mind, scrofula somehow seems to have developed a reputation that was markedly at odds with its biological reality. How did a condition of relatively low virulence, of which there is, according to modern studies, ‘no evidence of transmission ever from human contact’ become one of the period’s most prominent examples of pernicious hereditary disease?^7^

In this essay, I suggest that scrofula was one of several ostensibly related disorders, including gout, rickets, and venereal disease, that were ‘rebranded’ as hereditary in response to broader cultural changes that took place during the Restoration and eighteenth century. While the particular purposes of scrofula’s recategorisation were more political than medical, they resulted in this relatively obscure condition assuming a new significance within both the medical and popular discourses of the period. Scrofula went from being an unpleasant disorder that could afflict those of all ages to a deeply concerning sign—often emerging during the innocence of childhood—of inherited physiological damage.^8^ It served as both an emblem and proof of the links between sexual promiscuity, financial profligacy, and physiological degeneration, with its symbolic status reinforced by the legal and moral language used to model processes of hereditary transmission.^9^ By likening the inheritance of scrofula to the inheritance of original sin—or, more commonly, to the inheritance of a ‘docked entail’ or damaged estate—eighteenth-century writers not only made this minor ailment into a sign of catastrophic cultural and biological decline; they also paved the way for scrofula to be identified as a disease of the elite, even though its association with crowded, unsanitary living conditions likely made it more common among the poor. By the same token, financial models of disease inheritance facilitated a bias toward paternal transmission, with scrofula often (though not always) portrayed as passing, like a title or an estate, from father to son rather than from mother to daughter.^10^

As my timeline here suggests, I assert that scrofula’s reframing as hereditary took place some 150 years before the emergence of a distinct scientific vocabulary for biological inheritance. As Matthew Cobb explains, terms like ‘heredity’ and ‘genetics’ did not come into use until the early nineteenth century, when scientists began to reconcile theories of generational resemblance with information gleaned from medical reportage and agricultural practice.^11^ Perhaps for this reason, existing scholarly histories of heredity have often culminated in the nineteenth century, with work by Sean Quinlan, Carlos López-Beltrán, and others emphasizing the particular contributions of medical discourses in Revolutionary and Napoleonic France.^12^ In keeping with this line, Elizabeth Lomax has traced the debate over whether scrofula was a ‘hereditary or acquired disease’ back to French medical thought from the 1800s, citing Antoine Portal, professor of medicine at the Collège de France, as one early proponent of hereditary transmission.^13^

Yet scrofula’s recategorisation as hereditary began long before the nineteenth century in England. As work by Jenny Davidson, Victor Hilts, and others has shown, ideas about what would later be known as ‘heredity’ were already circulating in the early decades of the 1700s, with English commentators increasingly advocating what Quinlan terms ‘physical and moral hygiene’ to overcome the perceived dangers of inherited degeneracy and disease.^14^ With specific reference to scrofula, I suggest that ideas about hereditary transmission first rose to prominence during the Restoration, as physicians working in Charles II’s court sought to explain an alleged increase in the number of scrofulous cases. By the early 1700s, scrofula had already become, as Carlos López-Beltrán observes, ‘perhaps the constitutional disease that received most attention for its hereditary pattern’ within English medical works.^15^ Within popular print culture, it attained a complementary prominence, acting as a symbol of elite debauchery in attacks on aristocratic vice, fashionable arranged marriage, and royal misbehaviour. While these popular portrayals of scrofula as a disease of overindulgence were almost certainly inaccurate from an epidemiological perspective, they may have reinforced, and even prolonged, its continued miscategorisation as an inherited disorder.

## John Browne and the emergence of hereditary scrofula

As Quinlan observes, early modern physicians had long recognised that ‘the same diseases appeared repeatedly across family lines’, and treatises published in the sixteenth and seventeenth centuries identified a wide range of complaints—’syphilis, arthritis, phthisis, scrofula, rickets, gout, stones, epilepsy, and insanity’—as potentially hereditary.^16^ It was not until the Restoration of the monarchy in 1660, however, that scrofula began to attract particular attention in discussions of inheritance. Since the medieval period, scrofula had been known colloquially as ‘the king’s evil’—a name derived from the popular belief that the monarch’s touch could cure the disease. Proponents of the royal touch claimed that the first English king who possessed such healing powers was Edward the Confessor, but subsequent monarchs utilised this ‘hereditary gift’ to varying degrees, and the practice of touching for ‘the evil’ was discontinued altogether during the Interregnum.^17^

With the Restoration of the monarchy in 1660, ceremonies in which the king healed the scrofulous were revived, with extraordinary success: as Stephen Brogan explains, Charles’ return was greeted by an outpouring of ‘popular royalism’ that included renewed enthusiasm for royal therapeutics, and popular demand was so great that a regular schedule of ceremonies was quickly instituted, with sergeant surgeons appointed to preside over the procedure from a medical perspective.^18^ (Among other things, it was important to ensure that attendees really did have scrofula and were not merely seeking the valuable gold touch-pieces presented to supplicants). Charles II seems to have been as keen to promote the practice as his subjects were to receive it: Brogan observes that ‘greater numbers of people were touched for scrofula during the Restoration by both Charles II and James II, and with greater regularity, than at any other time in English history.’^19^

Whatever his motives for performing the touch, it seems likely that Charles II understood the propagandistic value of the ceremonies, as Marc Bloch and others have suggested.^20^ Not only did Charles II’s exercise of the ‘hereditary gift’ prove that he was the rightful heir to the throne; it also signalled his enjoyment of divine favour—perhaps a particularly valuable advantage for a monarch otherwise known for his rather *ungodly* behaviour at court. Certainly, the medical practitioners who assisted Charles II seem to have recognised an opportunity for self-advancement: it is no coincidence that the first two major treatises on scrofula were published during the Restoration. Both Richard Wiseman, as Sargeant Surgeon, and John Browne, as surgeon-in-ordinary, would have had a vested interest in drawing attention to the disorder and emphasising its prevalence and dangers, as well as endorsing the king’s powers as a healer.

According to Adam Komorowski and Sang Ik Song, Wiseman’s 1676 treatise constitutes a significant marker in the progress from thaumaturgic to medical healing, demonstrating ‘the contemporary evolution of the curative act away from royal prerogative towards the primacy of medical care’.^21^ Yet Wiseman’s colleague John Browne—dismissed by Komorowski and Song as a ‘plagiarist’—produced a treatise that arguably had far more lasting effects on popular beliefs about scrofula.^22^ When Browne’s *Adenochoiradelogia, or, An Anatomick-Chirurgical Treatise of Glandules and Strumæs* first appeared in 1684, it constituted the most extensive and detailed discussion of the disease to date, and its final book, on the ‘Royal Gift of Healing Strumæs [sic]’ remains the fullest surviving account of Charles II’s ceremonies to touch for the ‘evil’.^23^ Both medically and politically, Browne’s work was conservative, endorsing the efficacy of the king’s touch, and reproducing older humoral theories about the significance of diet and exercise in causing and treating the disease. Yet in the work’s second volume, Browne also advanced an important new theory about scrofula’s remote causes. The main intervention of this volume, *Chæradelogia*, was that it ‘introduced the Pox and Scurvy as two great Clubbers towards forming the [disease]’.^24^

While Browne stopped short of claiming that all cases of scrofula were hereditary, *Chæradelogia*’s strenuous emphasis on ‘Pox and Scurvy’ provided the catalyst for scrofula’s subsequent recategorisation as a sign of inherited degeneracy. Like most writers on hereditary disease in this period, Browne grappled with the question of whether disorders caused by an inherited predisposition or constitutional weakness should be considered hereditary.^25^ Although Browne concluded that cases in which ‘a Melancholy or intemperate Parent begets a Gouty Child, or a Strumous Brat’ could not ‘properly’ be labelled hereditary, such cases remained indicative of the close relationship between parental and childhood physiology:

This we have apparently seen, that although neither of the Parents were in their Infancy or childhood afflicted with this Strumous or Evil Disease, yet Symptomes thereof have suddenly shewn themselves, and their dispositions thereto in their Infants and Children; especially in such Parents who before Coition were evilly disposed by vitiosity of Body, or errour of Life, having enough in them to transmit and transplant the relation thereof to their issue.^26^

As this passage suggests, Browne identified vice—’vitiosity of Body, or errour of Life’—as the missing link between non-scrofulous parents and their scrofulous progeny. By tracing the disfigurement of the child back to the ‘errors’ of the parent, Browne made scrofula one more demonstration of all those Old Testament warnings about God ‘visiting the iniquity of the fathers upon the children to the third and fourth generation’.^27^ Attributing scrofula to ‘the Sins of the People’ was also politically prudent, aligning Browne’s work with royalist accounts of post-Interregnum England as a ‘sick body politic’ in need of royal healing.^28^

Browne’s treatise went on to connect scrofula with two diseases associated with ‘vitiosity of Body or errour of Life’: pox, which he described as the ultimate consequence of ‘living very high, and at ease’ and falling into ‘venery’; and scurvy, which he linked with ‘drinking too much wine’.^29^ These kinds of conditions did not simply punish the immediate overindulgence, Browne explained; they ‘polluted’ the blood for generations, producing ‘filthy Excrementitious Humours, corrupt Exulcerations, and the like’ that were then passed down ‘by way of Seminal Traduction, from Father to Son’.^30^ Once ‘the French Pox, Scurvy, Jaundies, Rickets, and the like’ were in the blood, they could affect the sinner’s offspring for generations.^31^ And since both pox and scurvy could ‘put on other Formalities’ and ‘disguise themselves under the Habits of several Diseases’, it could be difficult to discern any clear link between a venereal or scorbutic complaint in the parent and a scrofulous ulcer in the child.^32^

While Browne’s arguments for a ‘near affinity’ between scrofula, pox, and scurvy had no more evidential basis than his endorsements of the royal touch, his claims offered a plausible explanation for an alleged increase in scrofulous cases in Restoration England. Not only did the purported link between parental ‘venery’ or ‘debauch’ and childhood illness make sense of scrofula by modelling it as a kind of inherited sin, the slippage between different kinds of hereditary disease (pox, scurvy, gout, rickets, scrofula) helped explain observed phenomena that had puzzled other practitioners (like discrepancies between different generations or different siblings in the same family). The consideration that both pox and scurvy could be traced back to overindulgence also explained why rates of scrofula were on the rise: with greater opportunities for sexual and alcoholic enjoyment than ever before, most families in England were now ‘generally tinged more or less’ with some degree of scurvy or pox, Browne explained; no wonder, then, that scrofula too was rampant ‘in this our Age, where Debauch is so frequent, and Venery become the common Harlot of the Times.’^33^ The risk was heightened by increasing wealth and leisure time, as parental health was injured by ‘great intemperiety of Diet’ or by ‘an easie and sedentary life, stript from perplexities, anxieties, or vexations, as well as exercises, [and] seldom or never accustomed to labour’.^34^ Seen in this light, scrofula was not just a punishment for modern tendencies to sin; it was also a byproduct of emergent consumer capitalism, a disease that—to use Browne’s own terms—‘looks as if it got birth from Commerce and Conversation with Mankind; it arriving at that numerous Off-spring, that it meets us almost in every Street’.^35^

## Browne’s legacy: scrofula, sin, and spending

While Browne’s arguments for a link between scrofula and scurvy did not gain widespread acceptance, his theory about the correlation between scrofula and venereal disease was endorsed by many subsequent practitioners, from quacks peddling their cures for the ‘venereal taint’ to well-respected physicians addressing fellow professionals. Even as they dismissed the practice of the royal touch as ‘superstitious and insignificant’, physicians continued to reproduce Browne’s claim that the ‘two Evils, strumous and venereal, have such a Similitude and Relation to each other that a Transition from the last to the first is very easy, and…clearly exemplify’d in the sad Inheritance of Misery convey’d to Children by Parents who were long infected.’^36^ Unsurprisingly, practitioners who specialised in the treatment of venereal disease seem to have been particularly invested in these links: the surgeon Charles Peter was one of many proto-venereologists to claim that ‘the Pox in the Father, may prove the Rickets, Scurvy, Evil, an Asthma, or other Distemper in the Child.’^37^

Subsequent writers also echoed Browne’s claims about the links between scrofula and overindulgence, sometimes revamping the old humoral commonplaces against rich food and lack of exercise to point the finger of blame at England’s modern consumer economy. More than a century after the publication of Browne’s treatise, Hugh Smythson’s *The Compleat Family Physician* (1785) still attributed the rise of scrofula to ‘the dawn of commerce’, explaining that urbanisation and industrialisation had removed ‘the necessity of procuring the means of existence by violent exercise in pursuing the beasts of the forest, and by daily labour in cultivating the land for bread’.^38^ According to Smythson, it was not until the advent of commercial modernity that ‘this disease, the child of idleness, and the result of a sedentary life, first prevailed.’^39^

As Smythson’s moralizing terms perhaps suggest, the fact that these particular aspects of Browne’s treatise retained their currency for well over a century testifies not just to their ability to explain observed phenomena or justify a particular cure, but also to their coherence with wider cultural fears about the changing sexual mores and spending habits that came along with modernity.^40^ The terms of Browne’s account implicitly reinforced this link, drawing on commonplaces that likened the inheritance of illness to the inheritance of sin or debt. *Adenochoiradelogia* was one of many works from this period to describe scrofula as a ‘taint’, ‘pollution’, ‘corruption’, ‘filth’, or ‘impurity’ passed down from parent to child. Equally, Browne likened the inheritance of scrofula to the inheritance of damaged assets: when parents were sickly, he explained, children were as obliged to ‘share of their depraved Natures and Constitutions, as of their Estates and Fortunes’.^41^ While these comparisons—between illness and sin, health and wealth—did not originate with Browne, they implicitly reinforced his theories, and they remained the dominant models for understanding all kinds of hereditary disease throughout the eighteenth century. Subsequent practitioners likened not just scrofula, but rickets, gout, venereal disease, consumption, and other ostensibly hereditary disorders to an inherited ‘taint’, a damaged ‘estate’, or a ‘docked entail’.^42^ Some even invoked the comparative of the damaged family home or crumbling country seat. The physician and cold-bathing advocate John Floyer, for example, explained that untreated venereal disease resulted in ‘Ricketty, Kings-Evil’d, or Consumptive’ children because it ‘loosen[ed] some Rivets and Pins…that should go to the fastening of the Foundation of [a man’s] *Family*’.^43^ Floyer’s terms here are vaguely reminiscent of Biblical depictions of the body as a temple to God—but they specifically reference modern building practices, describing a house erected on a ‘Foundation’ and bound together by ‘Rivets and Pins’. Similarly, Richard Blackmore, the would-be epic poet and physician to William III, employed an edificial metaphor in describing the ‘Inheritance of Misery’ awaiting children whose parents’ bodies had been ‘demolished’, like so many derelict houses, ‘by venereal Sufferings’.^44^

## Scrofula and aristocratic vice in early eighteenth-century print culture

These comparisons between moral, financial, and physiological inheritance gave ‘the evil’ a new importance not just within the medical discourses of the period, but also within its popular print culture. As medical writers continued to debate scrofula’s nature, transmission, prognosis, and relation to other diseases, novelists, poets, and artists invoked the associations with sin and debt to link scrofula with a sexually and financially profligate aristocracy. In this context, hereditary scrofula quickly became a prominent symbol in what the historian Donna Andrew has described as a burgeoning eighteenth-century campaign against ‘aristocratic vice’.^45^ And unsurprisingly, the same moral and financial terms invoked in medical accounts also shaped imaginative representations of the disease, with the metaphors of the mortgaged estate, docked entail, or crumbling house encouraging imaginative characterisation of scrofula as a disease of the privileged instead of the poor.^46^

One writer who seems to have been particularly invested in these links is Jonathan Swift. In his 1708 satire *An Argument Against Abolishing Christianity*, Swift identifies scrofulous children as the unhappy consequence of sexual and financial profligacy among ‘men of wit and pleasure’. While the immediate context of Swift’s remarks is theological—he is querying whether the money spent on clergymen might be better used to ‘maintain, at least, two Hundred young Gentlemen of Wit and Pleasure, and Free-thinking’—his argument concludes with an attack on elite overindulgence.^47^ Defending Henry VIII’s seizure of church lands as salubrious for the populace, Swift’s speaker insists that enriching a group of ‘young Gentlemen’ would have damaged the nation’s health as well as its finances:

For, pray, what would become of the Race of Men in the next Age, if we had nothing to trust to, besides the scrophulous consumptive Productions furnished by our Men of Wit and Pleasure; when having squandered away their Vigour, Health, and Estates, they are forced, by some disagreeable Marriage, to piece up their broken Fortunes, and entail Rottenness and Politeness on their Posterity? Now, here are ten Thousand Persons reduced by the wise Regulations of *Henry* the Eighth, to the Necessity of a low Diet, and moderate Exercise, who are the only great Restorers of our Breed, without which the Nation would, in an Age or Two, become but one great Hospital.^48^

Here, Swift identifies ‘scrophulous, consumptive’ children as the unfortunate byproducts of elite ‘Pleasure’, invoking the same financial metaphors that populate contemporaneous medical texts to equate the ‘squandr[ing]’ of ‘Vigour’ and ‘Health’ with the squandering of ‘Estates’. In warning that debauched gentlemen ‘entail Rottenness and Politeness on their Posterity’ rather than land or money, Swift also links scrofula with sexual misbehaviour: sufferers of advanced venereal infections were often described as ‘rotten’, so there is a causal relation being implied between venereal disease in the parent and scrofula in the child.^49^ And although it is difficult to discern just how far the *Argument*’s irony extends, Swift’s critique here seems to move beyond theology, questioning the wider systems of wealth distribution and inheritance that favour the ‘scrofulous, consumptive Productions’ of the rich over their more robust but less privileged peers. ^50^ Hereditary diseases like scrofula, seen in this light, pose a threat not just to the elite, but to the whole ‘Race of Men in the next Age’.

Swift was to return to these issues again some twenty years later in *Gulliver’s Travels* (1726). Here, as in the *Argument Against Abolishing Christianity*, Swift attributes the degeneration of the species to the misbehaviour of ‘gentlemen’, linking hereditary scrofula with venereal disease.^51^ When Gulliver is visiting the island of Glubbdubdrib—a place in which the spirits of the deceased can be summoned to appear before the living—and asks to see ‘some of the modern dead, who had made the greatest figure, for two or three hundred years past, in our own and other countries in Europe’, he is unsurprised to see that ‘old illustrious families’ pass down their diseases alongside their privilege:

I confess it was not without some Pleasure that I found my self able to trace the particular Features, by which certain Families are distinguished up to their Originals. I could plainly discover from whence one Family derives a long Chin; why a second hath abounded with Knaves for two Generations, and Fools for two more; why a third happened to be crack-brained, and a fourth to be Sharpers….How Cruelty, Falshood, and Cowardice grew to be Characteristicks by which certain Families are distinguished as much as by their Coat of Arms. Who first brought the Pox into a noble House, which hath lineally descended in scrophulous Tumours to their Posterity. Neither could I wonder at all this, when I saw such an Interruption of Lineages, by Pages, Lacqueys, Valets, Coachmen, Gamesters, Fidlers, Players, Captains, and Pick-pockets.^52^

Here Gulliver surveys a wide range of ostensibly inherited features, moving from relatively benign flaws like ‘a long Chin’ to more serious failings like ‘Cruelty, Falsehood, and Cowardice’. His list culminates in the discovery of a venereal infection ‘which hath lineally descended in scrophulous Tumours to [a noble house’s] Posterity’. Once again, scrofulous children are identified as the consequence of parental misbehaviour, with tumours ‘lineally descend[ing]’ from one generation to the next like a title or estate. Swift’s phrasing plays cleverly on the multiple meanings of the word ‘house’, with the disease ‘brought in’ to a family’s bloodline much as a valued heirloom might be brought into the family home.

Further, while this passage seems to attribute the proliferation of undesirable characteristics to the ‘interruption of [aristocratic] lineages, by pages, lackeys, valets, coachmen, gamesters, fiddlers, players, captains, and pickpockets’, subsequent passages in *Gulliver’s Travels* suggest the reverse, with scrofula portrayed as a consequence of inbreeding *within* the nation’s upper classes. As Jenny Davidson and others have noted, Gulliver’s final voyage—to the Country of the Houhynhnms, an equine species possessed of superior intellectual abilities but highly questionable ethics—explores a number of unsettling possibilities for intervening in the breeding process.^53^ Not only do the Houhynhnms practice a system of controlled reproduction whereby the ‘inferior’ white, sorrel, and iron-grey horses are conditioned to believe it ‘monstrous and unnatural’ to ‘match out of their own Race’ by mating with bay, dapple-grey, or black breeds, the Houhynhnms also ‘rationally’ debate whether to perpetrate genocide against the vulgar and destructive humanoid ‘Yahoos’ with whom they share their land.^54^

In this context, Gulliver’s own intellect and appearance expose the wildly faulty premises on which the Houhynhnms’ eugenic reasoning is based: not only have these ‘rational animals’ assumed that qualities like beauty and intelligence are objectively definable absolutes that are inherited, but they have also assumed a direct correlation between desirable physiological or psychological traits and elevated social status. Thus, when one of the Houhynhnms, remarking on Gulliver’s superior appearance and intellect, concludes that he ‘must have been born of some Noble Family’, Gulliver must correct the assumption by explaining that the aristocracy has long since degenerated into disease and deformity through inbreeding:

I assured him…That, our young *Noblemen* are bred from their Childhood in Idleness and Luxury; that, as soon as Years will permit, they consume their Vigour, and contract odious Diseases among lewd Females; and when their Fortunes are almost ruined, they marry some Woman of mean Birth, disagreeable Person, and unsound Constitution (merely for the sake of Money), whom they hate and despise. That, the Productions of such Marriages are generally scrophulous, rickety or deformed Children; by which Means the Family seldom continues above three Generations, unless the Wife take Care to provide a healthy Father among her Neighbours, or Domesticks, in order to improve and continue the Breed. That, a weak diseased Body, a meager Countenance, and sallow Complexion, are the true Marks of *Noble Blood*; and a healthy robust Appearance is so disgraceful in a Man of Quality, that the World concludes his real Father to have been a Groom or a Coachman.^55^

Here, once again, ‘scrophulous, rickety, or deformed Children’ are traced back to the ‘odious Diseases’ contracted by a sexually incontinent elite. The terms of Swift’s account connect financial and sexual overindulgence, as he warns that those who are ‘bred from their Childhood in Idleness and Luxury’ will ‘consume their Vigour’ as well as their fortunes. And once again, the consequences of aristocratic vice ripple outwards, with noble families ‘seldom continu[ing] above three Generations’ unless the ‘Woman of mean Birth’ can find a non-aristocratic father with whom ‘to improve and continue the Breed’. Paradoxically, it is those of lower-class status—the wife and her male ‘Neighbours or Domesticks’—who possess the means of bettering their betters in this scenario.

## Scrofula and the mid-century critique of arranged marriage

Ultimately, *Gulliver’s Travels* attributes the rise in ‘scrofulous, rickety, or deformed children’ not just to the corrupt morals of the aristocracy, but also to the loveless marriages used to perpetuate aristocratic privilege. Swift’s views on this topic, as on the lure of eugenics, were eerily prescient: in the decades after the publication of *Gulliver’s Travels*, references to hereditary disease increasingly featured in critiques of elite arranged marriage, with some writers arguing that loveless unions encouraged adultery and thus facilitated the spread of venereal infections, while others highlighted the long-term dangers of aristocratic inbreeding as a cause of disease. In this context, hereditary scrofula seems to have served as something of a bellwether in the clash between ‘dynastic’ and ‘companionate’ marriage models, with popular references to the disease reaching their peak during the 1740s and 1750s, when the debates around ‘clandestine marriage’ (effectually marriage without parental consent) were at their height.^56^

Although many mid-century poems, novels, and plays expounded on the perils of loveless union, perhaps the best-known depiction of scrofula in this context appeared in a work of graphic art.^57^ In William Hogarth’s *Marriage A-la-Mode,* a set of six images first executed as paintings in 1742–43 and then reproduced as popular engravings from 1745 onwards, scrofula is depicted as one of several interconnected hereditary disorders—including gout, rickets, and venereal disease—that doom the loveless union between the son of a bankrupt earl and the daughter of a socially-aspiring alderman.^58^ In his advertisement for the series, Hogarth identified his subject matter as ‘Modern Occurrences in High-Life’, and *Marriage a-la-Mode* has often been interpreted as a critique of both aristocratic greed and middle-class ambition—but, as Sean Shesgreen observes, the work’s ‘exposure of the aristocracy is much more complete than its criticism of the [middle] class.’^59^ Like Swift, Hogarth blames hereditary scrofula on elite overindulgence, using the now-familiar metaphors of the mortgaged house and indebted estate to depict the disease as passing not just from father to son, but also from high to middling orders of society.

**Figure 1.**
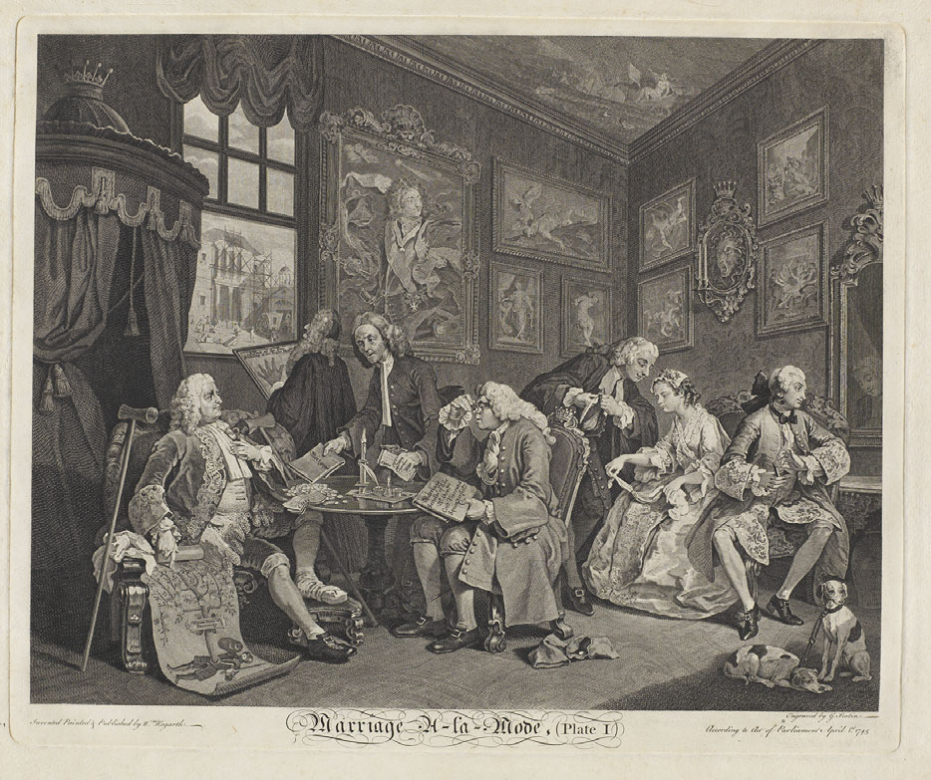
William Hogarth, *Marriage a-la Mode*, Plate 1: ‘The Marriage Settlement.’ Reproduced courtesy of the Lewis Walpole Library, Yale.

To that end, the first plate’s bipartite composition seems to draw attention to class and generational divides: on the right-hand side of the print, the would-be bride and groom sit facing away from each other in a sign of their mutual disregard; on the left, their fathers draw up a marriage contract that serves their own ambitions (Figure 1).^60^ While the bride’s father, an upwardly mobile merchant, pursues the debatably less selfish goal of obtaining a title for his daughter, the groom’s father, a louche aristocrat rather unsubtly named Lord Squander or Lord Squanderfield, seeks to fund his profligate lifestyle. The Earl’s lavishly furnished home, gouty foot, and fashionable clothing all identify him as a recognisable type: he is one of those ‘men of wit and pleasure’ condemned by Swift, a spendthrift aristocrat who has squandered his wealth on needless extravagances, and who must now ‘piece up [his] broken fortunes’ by arranging a marriage of convenience for his son.^61^

**Figure 2.**
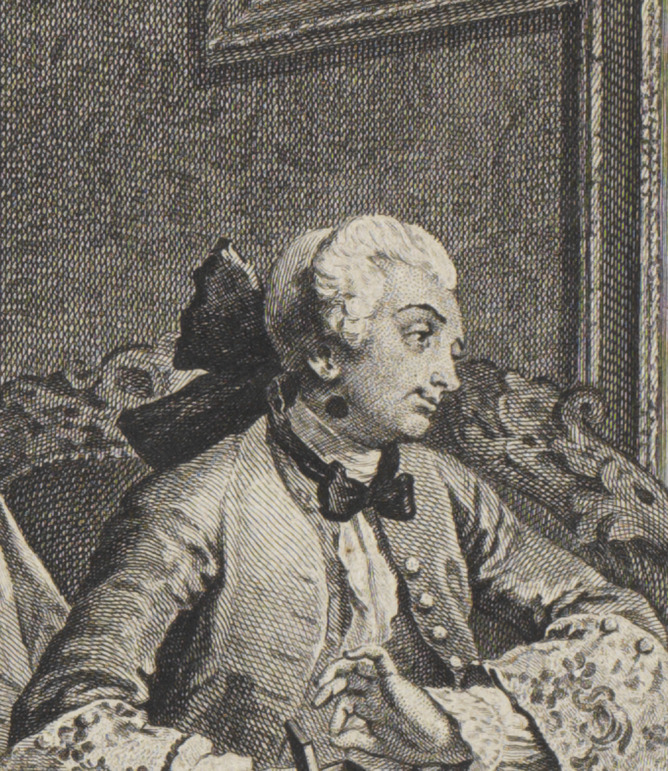
Detail from *Marriage a-la-Mode*, Plate 1: ‘The Marriage Settlement.’ Reproduced courtesy of the Lewis Walpole Library, Yale.

Although the Viscount’s preening posture and showy clothing suggest that he shares his father’s appreciation for the finer things, other aspects of his appearance warn that he will be forced to pay, physiologically and financially, for his father’s sins. His spindle shanks are suggestive of physical frailty, and as he turns to admire his reflection in a mirror, he exposes a large black patch on his neck (Figure 2). While often interpreted by critics as a sign of venereal infection, this patch is of a larger size than other venereal ‘beauty spots’ in Hogarth, and its placement on the Viscount’s body strongly suggests it conceals not a syphilitic sore but a scrofulous ulcer.^62^ The Viscount does, admittedly, contract a venereal infection later on in the series—but in this first plate, just as the Earl’s gout, clothing, and surroundings identify him as one of those ‘men of wit and pleasure,’ so this black patch identifies the Viscount as one of the ‘scrofulous, consumptive productions’ of the misbehaving elite.

Mirroring and duplication further situate both the Earl and the Viscount within a longer line of degenerating Squanderfields. While the Earl’s surroundings implicitly attribute his gout to epicurean overindulgence, both gout and scrofula were believed to be hereditary diseases, so the Earl’s condition, too, may descend from prior generations. Certainly, the portrait that hangs on the wall behind him suggests an element of generational transfer, as the ancestor staring confidently out from the frame looks like the present Earl, but is, as Frederick George Stephens explains, ‘a general officer of Marlborough’s time’, for he ‘wears over his cuirass the ribands of two orders, one of which is the Golden Fleece’.^63^ According to Ronald Paulson, Judy Egerton, and others, the man in the portrait *is* the present Earl, and thus the Fleece—’an honour awarded to no Englishman between Henry VIII and the Duke of Wellington’—is an anachronism, exposing the Squanderfields as modern upstarts; yet the resemblance between the painted ancestor and the present Earl makes perfect sense in relation to the work’s wider themes of generation, regeneration, and degeneration. ^64^ Just as the scrofulous Viscount takes after his gouty father, so the rapacious Earl takes after an unnamed ancestor whose posture signals rapacity of a different kind: he stands with legs akimbo, a canon firing beside him at crotch height. Here, then, as in Swift’s satire, hereditary diseases like gout and scrofula are ultimately traced back to sexual profligacy—and, likely, sexual disease (the canon may be a reference to the ‘fire’ of venereal infection)—in a male ancestor.

**Figure 3.**
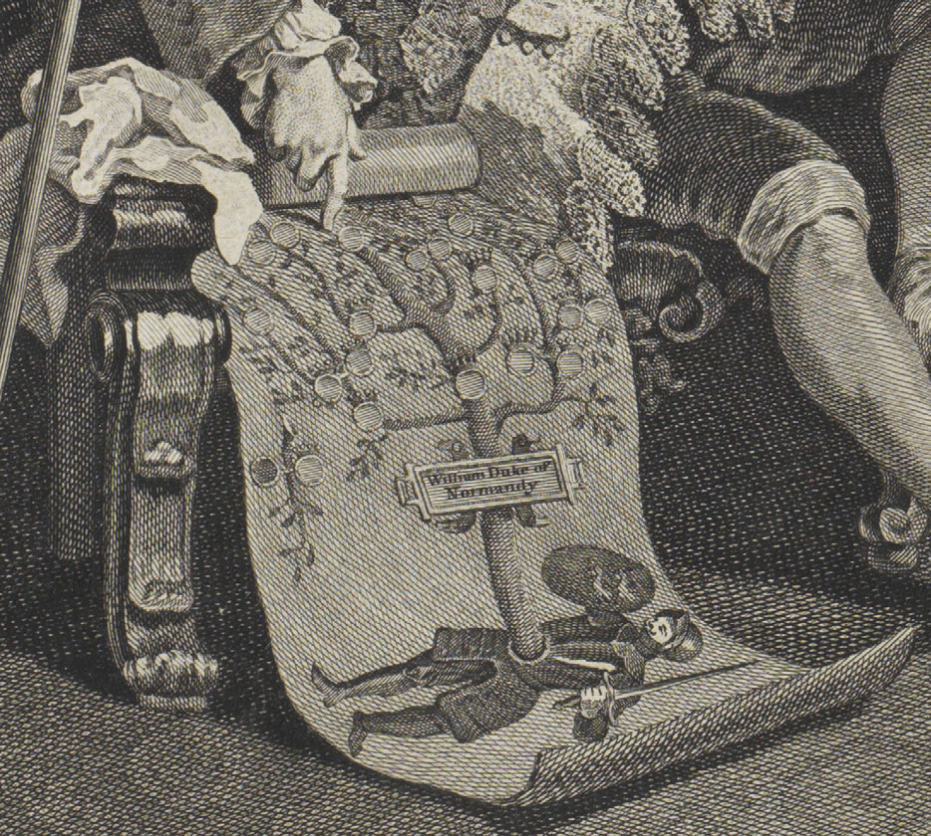
*Marriage a-la-Mode*, Plate 2: ‘The Tête à Tête’. Reproduced courtesy of the Lewis Walpole Library, Yale.

Another detail in the plate also implicitly links the viscount’s scrofula with familial inheritance. As the gouty Earl urges the alderman to an agreement, he points to a large family tree he has produced as proof of his son’s illustrious lineage. This scroll traces the Squanderfield family back to the Duke of Normandy—but it also reveals a doomed, childless union between some prior Squanderfield and an ignominious outsider, with one branch of the tree ending abruptly in a link between a white spot (presumably indicating one of the family) and a black spot (presumably indicating a shameful connection). Whether this black spot is understood as a prediction of the family’s future or a record of the past, its presence creates a visual echo with the black spot on the young Viscount’s neck, linking the ill-fated Squanderfield line with the transmission of hereditary scrofula. (Figure 3).

Finally, Plate 1 makes ingenious use of financial and edificial metaphors, paralleling contemporary textual representations of hereditary diseases like scrofula, gout, and venereal disease. The usurer who stands between the two patriarchs simultaneously collects the merchant’s banknotes in payment of the Earl’s outstanding debts and presents the Earl with a new mortgage—an agreement presumably arranged to finance the completion of the half-finished family mansion just visible outside the window. The new Squanderfield home is at once extravagant and uninhabitable: while grand in scale and ostentatious in design, it remains unfinished, its empty scaffolding indicative of construction halted by a lack of funds. These details suggest that, much like those sickly children who succeed to the ‘docked entail’ of an unsound constitution, the scrofulous Viscount will inherit a half-finished and heavily-mortgaged estate, a house of impeccable pedigree (a fashionable architect stands at the window holding ‘A plan of the New Building of the Right Hon[ora]ble’) but very uncertain stability.

**Figure 4.**
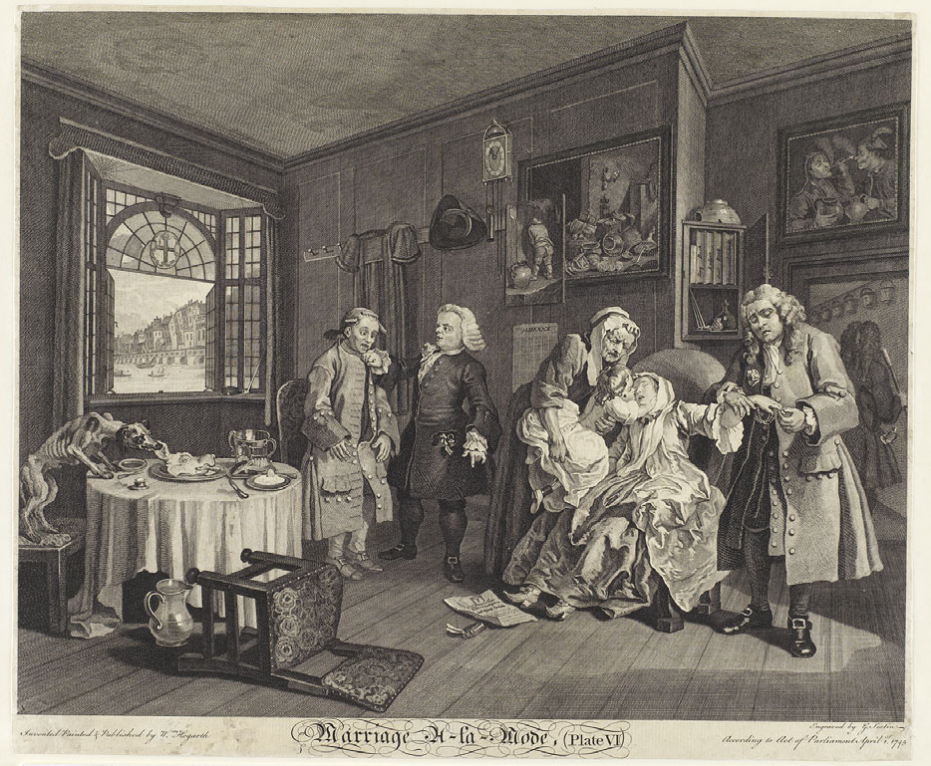
Marriage a-la-Mode, Plate 3: ‘The Inspection.’ Reproduced courtesy of the Lewis Walpole Library, Yale.

**Figure 5.**
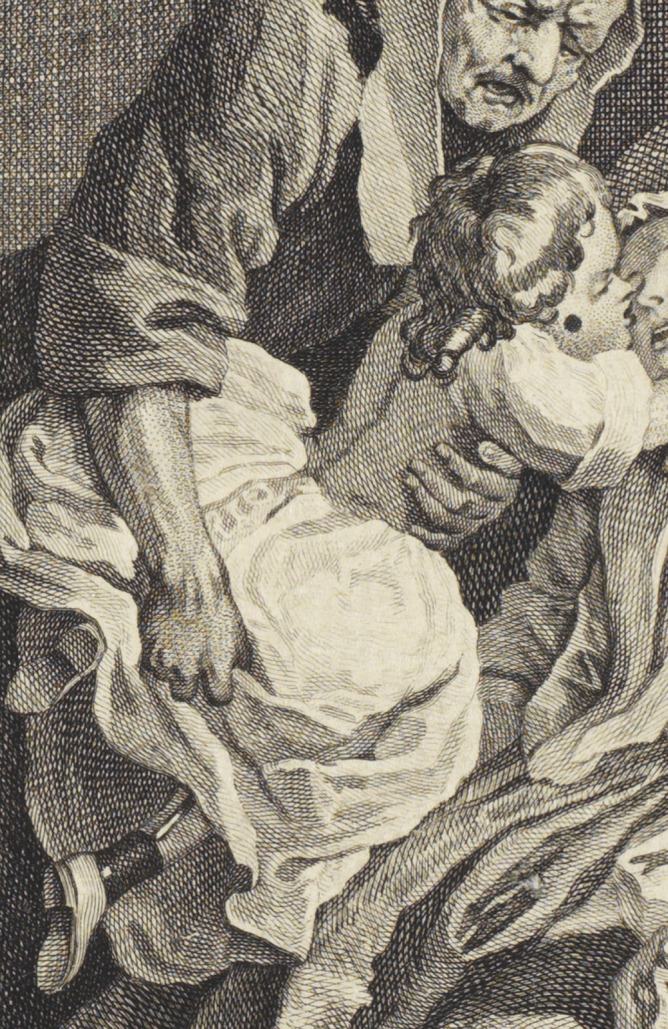
Marriage a-la-Mode, Plate 6: ‘The Lady’s Death.’ Reproduced courtesy of the Lewis Walpole Library, Yale.

These same metaphors recur in the sixth and final plate in the series, as the fashionable marriage of the title reaches its disastrous conclusion. By this point, both the Viscount, now Earl, and his wife, now Countess, have met with unnatural ends: the Earl has been fatally stabbed by his wife’s lover, and the Countess has taken her own life in despair at her lover’s execution for the murder (Figure 4). The focal point of this final plate, however, is the sickly infant being pulled from the dead Countess’ arms. Here, then, is the heir to the Squanderfield (mis)fortunes, a child whose grim physical condition signals its miserable ‘docked entail’ of hereditary illness.^65^ The child’s legs, deformed by rickets, are supported with metal braces beneath its clothing, and its cheek features a large black patch covering a scrofulous sore just like its father’s (Figure 5, detail). Once again, physiological degeneration is translated into financial and edificial terms, as the child is pictured not in the grand Squanderfield mansion visible outside the window in Plate 1, but rather, in the dark, inhospitable home of its miserly maternal grandfather. The room’s bare floors and broken windowpanes not only mark the Squanderfield inheritance as punitive and insalubrious; they also render in literal terms the familiar cautionary tale of morally and financially ruined ‘houses’.

While *Marriage a-la-Mode* was among the less successful of Hogarth’s ‘Modern Moral Subjects’, many mid-century texts echoed its depiction of the pathological consequences of arranged marriage, with some writers explicitly mentioning hereditary scrofula. ^66^ In the 1749 satire *A Journey from This World to the Next*, for example, the novelist Henry Fielding—a longstanding admirer of Hogarth’s work—followed *Marriage a-la-Mode* in attributing an alleged increase in hereditary diseases like scrofula to the fashion for arranged unions among the elite. In one episode from this eccentric mock-travelogue, Fielding’s narrator—a newly-deceased spirit who is travelling through the underworld—encounters a ‘French Lady’ named ‘Maladie Alamode’: a personification of venereal disease whose name plays on contemporary terms for the ‘French pox’ as the ‘a la mode disease’, or the ‘fashionable distemper’.^67^ Maladie is introduced as a woman of consequence, and when she welcomes the narrator into her enormous underworld palace, she explicitly attributes her success to the fashion for loveless unions among those of ‘considerable rank’:

She spoke likewise greatly in approbation of the method so generally used by parents, of marrying children very young, and without the least affection between the parties; and concluded by saying, that if these fashions continued to spread, she doubted not, but she should shortly be the only disease who would ever receive a visit from any person of considerable rank.^68^

Here, as in Hogarth’s series, arranged marriage is portrayed as facilitating infidelity, and thus the spread of venereal infections, among the elite. When Maladie goes on to boast of her ability to provide for her three ‘daughters’, Lepra (leprosy), Chaeras (scrofula), and Scorbutia (scurvy), Fielding’s witty use of personification renders literal the symbolic conception of venereal disease as the progenitor of all hereditary diseases. Similarly, Maladie’s complaint that all three of her daughters ‘had the confidence to deny themselves to be her children’ renders in imaginative terms the medical theories that scrofula, pox, and scurvy could manifest differently in different generations.^69^ Although Fielding’s gendering of all four diseases as female is highly unusual, his satire exemplifies the continuing popular conviction that hereditary scrofula was a consequence of parental venereal disease.

Similar concerns about the physiological consequences of aristocratic arranged marriage continued into the 1750s, with some writers explicitly invoking the dangers of hereditary scrofula to protest against Hardwicke’s 1753 Marriage Act. This piece of legislation, designed to prevent young lovers from marrying without parental consent, was viewed by many commentators as a deliberate attempt to prevent unions between those of disparate class or financial backgrounds.^70^ And some opponents of the bill, including the physician-turned-writer John Shebbeare and the Tory propagandist Arthur Murray, warned that the Act was not just a strike against marital harmony; it was also a recipe for biological disaster. In a February 1754 issue of the Tory periodical *The Gray’s Inn Journal*, for example, Murray imagined a dystopian future in which the Marriage Act had accomplished the ‘Ruin of our British Nobility’, with the most powerful families weakened and debilitated by years of legally sanctioned inbreeding. ‘I already see them dwindling by Degrees into an emaciated, puny, sickly Race,’ he warned, ‘and let me tell you…it cannot be otherwise if, for the future, they are only to marry among one another, without any possible Means of improving the Breed by a Mixture of wholesome Flesh and Blood.’^71^ Murray goes on to imagine the ‘News for one Hundred Years Hence,’ with his society pages identifying the ‘tallest Nobleman in England’ as ‘near four Feet and ten Inches’, and describing another gentleman as forced to contract an ‘advantageous Match’ with a ‘Miss *Ricketty Barren*’ instead of a love marriage ‘with Miss *Maria Healthy*, a beautiful young Lady of moderate Fortune’.^72^ He reports on two sickly aristocrats, ‘Lords Scrophulous and Spindle’, injured by a carriage accident in which ‘both their Heads were unfortunately knocked together.’ ‘Very happily,’ he concludes, ‘their Lordships received no great Hurt,’ thanks to an inherited absence of brains.^73^ Although Murray’s work, like Fielding’s, is strikingly atypical in some respects—most notably in its silence about venereal disease, with inbreeding rather than infidelity identified as the chief danger of arranged marriage—the ‘News for One Hundred Years Hence’ reinforces once again the links between arranged marriage and a whole cluster of ostensibly related hereditary disorders, including scrofula, spindle shanks, infertility, and rickets.

## Scrofula in the late eighteenth century and beyond

While hereditary scrofula gradually lost its symbolic prominence in the final decades of the eighteenth century, both medical and non-medical writers continued to associate the disease with venereal infection or sexual misbehaviour, as well as to assert its potential for hereditary transmission. Although practitioners did highlight other possible causes for the disease, they also continued to understand it in relation to what we would now term heredity, debating whether scrofula itself, or some constitutional weakness that predisposed a child to scrofula, could be inherited. Popular medical manuals simplified these debates considerably but retained the focus on inheritance, with late-century editions of *Culpeper’s English Physician*, for example, explaining that scrofula ‘proceeds often from an hereditary taint’, and William Buchan’s *Domestic Medicine* (1772) advising that ‘Children who have the misfortunate to be born of sickly parents, whose constitutions have been greatly injured by the French-pox, or other chronic diseases, are apt to be affected with the scrophula.’^74^

Relatively few imaginative works mentioned scrofula in the latter half of the century, but those that did often continued to use it as a metaphor for aristocratic vice or consumerist excess. Charles Churchill’s 1764 satire *The Times*, for example, advised wealthy parents to spread the rumour that their son is already ‘scarr’d / With the Small Pox’ and ‘Eat up with the Kings-evil, and his blood, / Tainted throughout, a thick and putrid flood’.^75^ Churchill’s friend and former schoolfellow William Cowper also invoked the links between scrofula and aristocratic vice some twenty years later, in his long poem *The Task* (1785):

Increase of pow’r begets increase of wealth;

Wealth luxury, and luxury excess;

Excess, the scrofulous and itchy plague

That seizes first the opulent, descends

To the next rank contagious, and in time

Taints downward all the graduate scale,

Of order, from the chariot to the plough.^76^

Here, Cowper uses scrofula as a metaphor for ‘excess’ among the ‘opulent’, with increases in ‘Wealth’ and ‘luxury’ resulting in the physiological as well as moral corruption among the upper ranks. And like Swift or Hogarth, Cowper warns that scrofulous corruption rarely ends with the elite: it ‘Taints downward’ through the class hierarchy as well as through the generations, ultimately posing a risk to the whole ‘Race of Men in the next Age’.

Scholars have already examined how the eighteenth- and nineteenth-century medical discourses around hereditary disease may have anticipated the later fields of genetics and eugenics, so I want to conclude this essay with one final illustration of how popular print culture continued to invoke the ‘moral panic’ over hereditary disease at the end of the century.^77^ In the 1780s and 1790s, the future King George IV was known for his financial and sexual profligacy, routinely spending beyond his means and engaging in a series of high profile love affairs while still a bachelor. The satires that emerged to attack George’s debauchery routinely referenced hereditary disease as both a cultural symbol and a real-life threat, depicting damaged or disfigured heirs as the inevitable consequences of the prince’s imprudent behaviour. Ironically, where royalists like John Browne had once presented the ‘Sins of the People’ as the ‘great procurer’ of scrofula and the king’s touch as its cure, critics of George’s bachelor lifestyle warned that this future monarch was more likely to *cause* the evil than cure it.^78^

**Figure 6.**
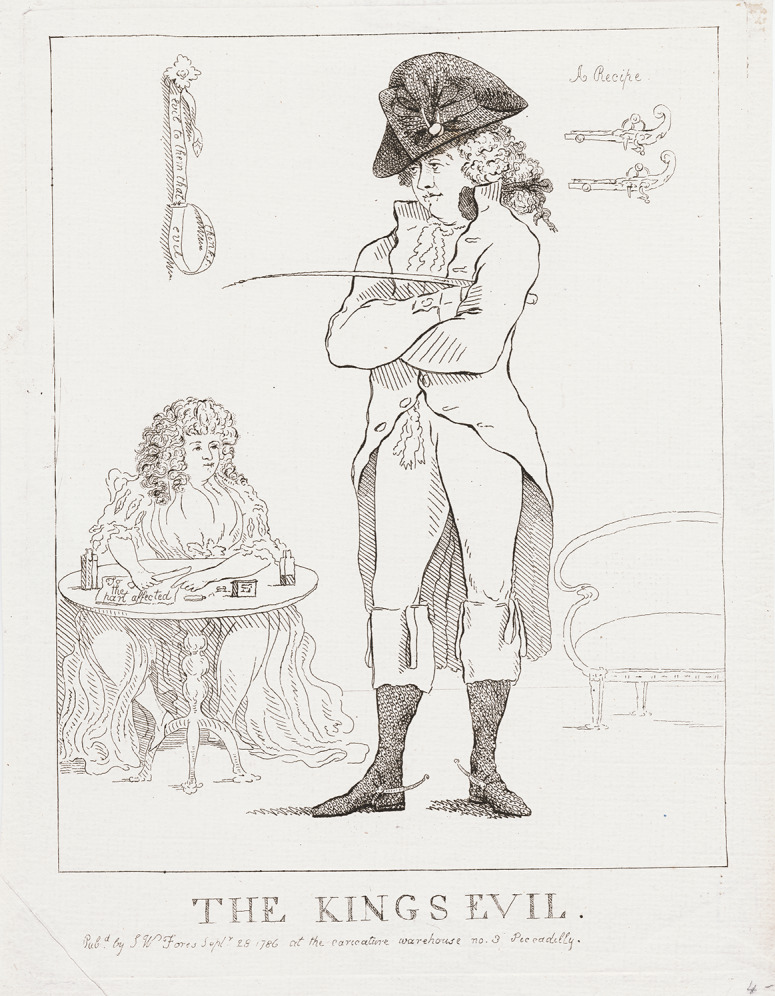
Anon., *The King’s Evil* (1786).

As I have noted elsewhere, dozens of satiric prints from this period attacked the prince’s sexual behaviour by implying that he or his mistresses had contracted a venereal disease.^79^ Some of these satires also emphasised the particular dangers of hereditary degeneration, linking George’s behaviour not just with the immediate embarrassments of the pox, but also with the long-term consequences of diseased or deformed children. In the anonymous 1786 print *The King’s Evil,* for example, George’s scandalous affair with an older Catholic widow named Maria Fitzherbert is the catalyst for an attack that links the prince’s sexual misbehaviour with both a venereal infection and the ‘evil’ of the title (Figure 6). By the time this print appeared, George and Fitzherbert had been lovers for over two years, but matters were approaching a crisis point: with the king’s health failing and the prince increasingly likely to step in as regent, pressure was mounting for the prince to forgo his profligate ways and abandon Fitzherbert—ineligible for the role of royal spouse on the grounds of her Catholicism and her widowhood—to find a suitable wife. Like many satires on the prince and Fitzherbert during this period, *The King’s Evil* links immorality with disease, implying that the prince’s sexual misbehaviour will have pathological as well as political consequences.

The image depicts George and Fitzherbert in a sparsely furnished room, with George standing in the foreground and his mistress seated behind him, her legs spread apart in a blunt visual signal of her sexual availability. The bottle of medicine that sits on the table before Fitzherbert, labelled with instructions to apply it ‘to the part affected’, signals the sexual and pathological nature of her relation with the future king. George stands before his lover in a pair of tight-fitting breeches, with Fitzherbert’s sightline terminating at the ‘part’ to which she must apply this newly purchased remedy. There is a clear suggestion here of shared venereal disease, not only in Fitzherbert’s posture and the medicine bottle, but also in the two pistols mounted upside down on the wall behind George: their presence invokes the literary commonplace of the penis as weapon, implying that the prince’s ‘guns’ are disabled by infection.

While the spectre of venereal disease looms large in this image, both the presence of the mismounted guns and the pun of the title draw attention to the dangers of George’s behaviour for the future of the royal line. In labelling this image ‘The King’s Evil’, the artist leaves open multiple interpretive possibilities: on one hand, the title can be understood as a straightforward attack on Fitzherbert, identifying her as an ‘evil’ afflicting the future king. On the other hand, however, the title could reference the dangers of George’s behaviour for his line of succession, punning on the popular name for a disease still widely understood as a sign of inherited degeneracy. Either way, *The King’s Evil* ridicules the prospect of thaumaturgic healing, contrasting the profligate behaviour of the prince with the healing powers of Edward the Confessor. Where once the evil was so named because the king was thought to cure it, now the evil is so named because the king’s heirs will have it.

**Figure 7.**
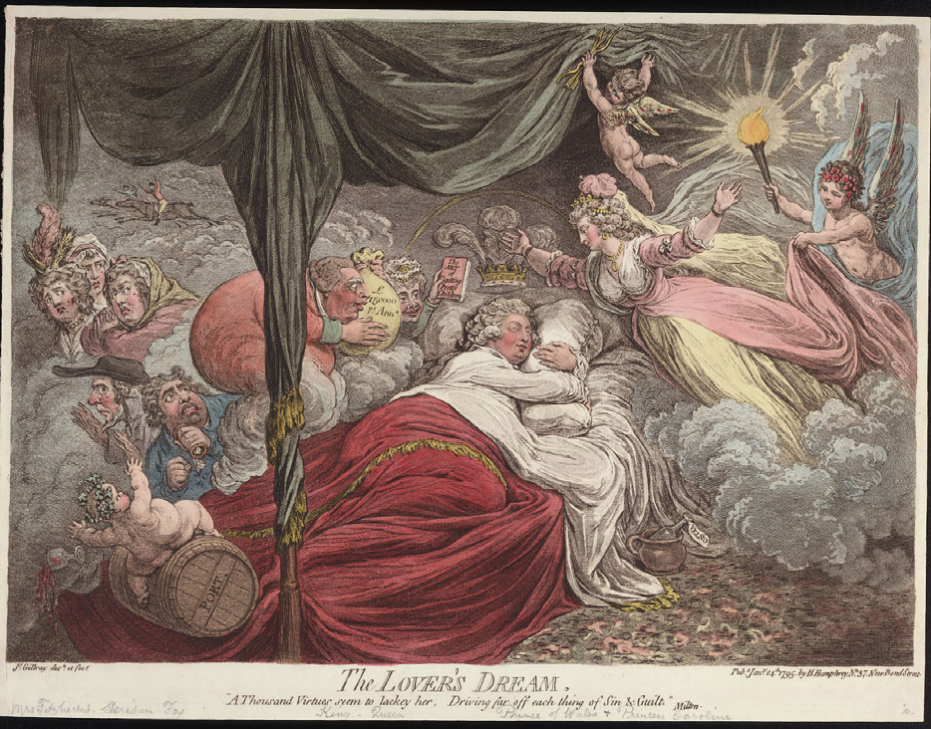
James Gillray, *The Lover’s Dream* (1795). Reproduced courtesy of the Lewis Walpole Library, Yale.

Other satires from the period make similarly allusive references to the potential pathological consequences of George’s misbehaviour. Those prints that depict the prince in possession of the popular venereal disease remedy Velno’s Vegetable Syrup, for example, take on a new valence when we consider that Velno’s was also touted as a cure for ‘Scorbutic, Scrophulous, Leprous, Rheumatic, and Paralytic Cases’.^80^ In one such image—James Gillray’s 1795 satire *The Lover’s Dream*—the prince is clearly condemned not just for his past profligacy, but also for the ‘taint’ of hereditary disease that his indiscretions risk introducing into the royal bloodline. By the time Gillray’s print appeared in 1795, the prince had ostensibly abandoned Fitzherbert and agreed to what would ultimately prove a disastrous marriage to his cousin, Caroline of Brunswick. In Gillray’s image, George is pictured asleep in a plush and comfortable bed: corpulent and ruddy-cheeked, he smiles as tantalising dreams of his privileged future life appear before him. (Figure 7).^81^ A beautified vision of Caroline holds pride of place, her arms outstretched to welcome the sometime prodigal into the absolving bonds of marriage; beside her, King George III and Queen Charlotte smile their parental approval of this Hogarthian marriage a-la-mode, the king holding out a bag labelled ‘£150000 p[e]r Ann[um]’—the amount to be settled on the prince in return for his cooperation.

**Figure 8.**
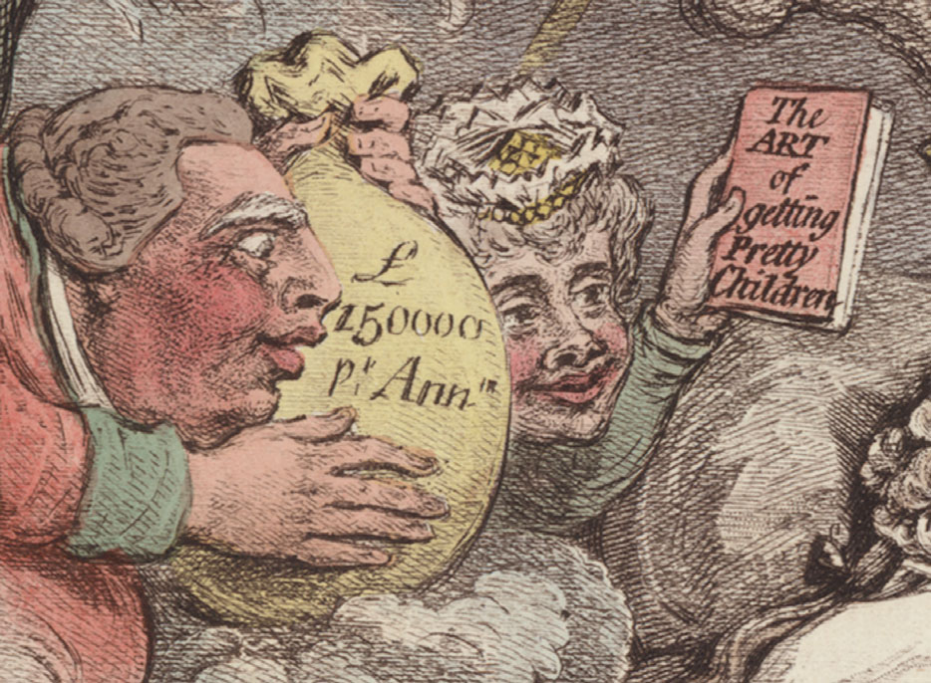
Detail from *The Lover’s Dream.* Reproduced courtesy of the Lewis Walpole Library, Yale.

Other details in the print, however, including the bottle of Velno’s Vegetable Syrup on the floor beside the prince’s bed, warn that George’s future will not be so untroubled as his dream suggests. To wit, while the king holds out a gift of wealth, the Queen proffers a book labelled ‘The Art of Getting Pretty Children’ (Figure 8). Here Gillray references Claude Quillet’s *Callipædia: Or, The Art of Getting Pretty Children*, a popular poem on how to engineer ‘a Race more noble and refin’d’ through selective breeding (or, as we might now term it, a eugenics manual).^82^ Quillet’s poem, first published in 1655 and reprinted many times over the course of the century, both in its original Latin and in English translations, was emphatic about the need to breed out hereditary diseases, warning that ‘those, whom eager Fires and latent Sores / Corrode’ were fit only ‘for the Bed of Sickness, not of Love’.^83^ While Quillet’s cautions against ‘Sores’ and ‘Ulcers’ are oblique from a diagnostic perspective, there is no mistaking the presence of *Callipædia* in Gillray’s satire: here, as in *Gulliver’s Travels* or *Marriage a-la-Mode*, elite misbehaviour is identified as a source of degeneracy, a risk to the health—even survival—of the royal line. (Indeed, Quillet’s text warns that evils ‘Oft reach to Kings, and o’er the Throne prevail;/ From a distemper’d Monarch’s lazy Loves’.)^84^ And once again, financial and physiological corruption are linked, with the parental wedding gifts acting as parallel talismans: against ruined health on the one hand, and ruined finances on the other.

While literary and visual depictions of scrofula changed considerably over the course of the nineteenth and twentieth centuries, the unique cultural prominence accorded to hereditary scrofula in eighteenth-century English print culture ultimately constitutes more than just a misconceived anticipation of future genetic or eugenic theories; it also offers a salutary reminder of how the metaphors used to conceptualise a disease can influence theories about its ætiology, transmission, and treatment. By likening the inheritance of scrofula to the inheritance of sins or debts, medical and popular discourses gave this relatively obscure disorder a set of lasting and influential meanings; its prominence in the print culture of the eighteenth century evidences not just a developing interest in physiological inheritance, but also an unsettling tendency to align medical diagnoses with moral and financial ones.

## Footnotes

1

Richard Wiseman , Severall Chirurgicall Treatises (London: E. Flesher and J. Macock, 1676), 249 .

2

Thomas White , A Treatise on the Struma or Scrofula, Commonly Called The King’s Evil. (London: J. Murray, 1784), ix.

3

Thomas White , *A Treatise on the Struma or Scrofula.* 2nd edition (Lo^nd^on: J. Murray, 1787), 3.

4 The pathogenic link between scrofula and tuberculosis was not identified until the early nineteenth century, and even then, as Elizabeth Lomax has explained, many physicians ‘regarded scrofula and tuberculosis as distinct diseases’. (See E. Lomax , ‘Hereditary or Acquired Disease? Early Nineteenth Century Debates on the Cause of Infantile Scrofula and Tuberculosis’, Journal of the History of Medicine and Allied Sciences, 32, 4 (1977), 356–74, 357336687 10.1093/jhmas/xxxii.4.356). See also Clark Lawlor , Consumption and Literature: The Making of the Romantic Disease (Basingstoke: Palgrave, 2006), 172–3.

5 R.P. Belin, J.D. Richardson, D.L. Richardson, *et al.* ‘Diagnosis and Management of Scrofula in Children’, *Journal of Pediatric Surgery,* 9, 1 (1974), 103–7; Jenish Bhandari and Pawan K. Thada, ‘Scrofula’ in *StatPearls.* Available at https://www.ncbi.nlm.nih.gov/books/NBK557700/. Accessed 24 November 2022.

6 See P. Zimmermann , M. Tebruegge , N. Curtis , et al. ‘The Management of Non-Tuberculous Cervicofacial Lymphadenitis in Children: A Systematic Review and Meta-Analysis’, Journal of Infection, 71, 1 (2015), 9–18 25727993 10.1016/j.jinf.2015.02.010.

7 Belin *et al.*, ‘Diagnosis and Management’, *op. cit.* (note 5), 103–7.

8 While medical texts from this period often focused on scrofulous children when highlighting the particular dangers of hereditary infection, it is worth emphasising that scrofula could persist into or begin in adulthood and that many of those who sought treatment in this period were adults.

9 On legal inheritance as a model for heredity, see Staffan Müller-Wille and Hans-Jörg Rheinberger, *A Cultural History of Heredity* (Chicago: University of Chicago Press, 2012), 5–6, 45–52.

10 One notable exception to this—appearing in both medical and popular texts (like Richardson’s *Pamela*)—is the alleged transmission of scrofula via tainted breast milk.

11

Matthew Cobb , ‘Heredity Before Genetics: A History’, Nature Reviews, 7, 12 (2006), 953–810.1038/nrg194817139326. See also François Jacob, The Logic of Life: A History of Heredity (Pantheon: Random House, 1973); Müller-Wille and Rheinberger, *Cultural History*, *op. cit.* (note 9), esp. 41–69.

12 See, for example, Sean Quinlan, *The Great Nation in Decline: Sex, Modernity and Health Crises in Revolutionary France*, c. 1750–1850 (Burlington: Ashgate, 2007); S. Quinlan , ‘Heredity, Reproduction, and Perfectibility in Revolutionary and Napoleonic France, 1789–1815’, Endeavour, 34, 4 (2010), 142–5020947167 10.1016/j.endeavour.2010.09.003; Carlos López-Beltrán , ‘“Les maladies héréditaires”: Eighteenth-Century Disputes in France’, Revue d’Histoire des Sciences, 48, 3 (1995), 307–5011609190; Mary Terrall , ‘Material impressions: conception, sensibility, and inheritance’, in H. Deutsch and M. Terrall (eds), Vital Matters: Eighteenth-Century Views of Conception, Life, and Death (Toronto: University of Toronto Press, 2012), 109–129 . Many historians of heredity cite the essay competitions on hereditary disease issued by French institutions (the Académie des Sciences in Dijon in 1748, the Société Royale de Médecine in Paris in 1788 and 1790) as the impetus for later developments.

13 Lomax, ‘Hereditary or Acquired’, *op. cit.* (note 4), 356–74.

14 Quinlan, *Great Nation*, *op. cit.* (note 12), 4. See also Sean M. Quinlan , ‘Inheriting Vice, Acquiring Virtue: Hereditary Disease and Moral Hygiene in Eighteenth-Century France’, Bulletin of the History of Medicine, 80, 4 (2006), 649–7617242550 10.1353/bhm.2006.0141; Jenny Davidson , Breeding: A Partial History of the Eighteenth Century (New York: Columbia, 2009); Victor Hilts , ‘Enlightenment Views on the Genetic Perfectibility of Man’, in Everett Mendelsohn (ed.), Transformation and Tradition in the Sciences: Essays in Honor of J. Bernard Cohen (Cambridge: Cambridge University Press, 1984), 255–71; Carlos López-Beltrán , ‘Forging Heredity: From Metaphor to Cause, a Reification Story’, Studies in History and Philosophy of Science, 25, 2 (1994), 211–3511639899 10.1016/0039-3681(94)90028-0; Carlos López-Beltrán, ‘Les maladies héréditaires’, *op. cit.* (note 12), esp. 321–6; Staffan Müller-Wille , ‘Figures of inheritance, 1650–1850’, in Staffan Müller-Wille and Hans-Jörg Rheinberger (eds), Heredity Produced: At the Crossroads of Biology, Politics, and Culture 1500–1800 (Cambridge: MIT Press, 2007), 177–204

15 Carlos López-Beltrán, ‘Forging Heredity’, *op. cit.* (note 14), 223.

16 Quinlan, ‘Inheriting Vice’, *op. cit.* (note 14), 654.

17 There was a parallel belief in France, with Philip I (1052–1108) believed to be the first king able to cure scrofula with his touch.

18

Stephen Brogan , The Royal Touch in Early Modern England: Politics, Medicine and Sin (Woodbridge: Boydell, 2015), 102 .

19 Brogan, *Royal, op. cit.* (note 18), 99. See also David Sturdy , ‘The royal touch in England’, in H. Duchhardt and R.A. Jackson (eds), European Monarchy: Its Evolution and Practice from Roman Antiquity to Modern Times (Berlin: Verlag, 1992), esp. 174–80.

20 See Marc Bloch , The Royal Touch: Sacred Monarchy and Scrofula in England and France, trans. J. E. Anderson (London: Routledge & Kegan Paul, 1973), 110, 211–2; Keith Thomas , Religion and the Decline of Magic: Studies in Popular Beliefs in Sixteenth and Seventeenth Century England (London: Weidenfeld and Nicolson, 1971), 195–6, 204–6; James F. Turrell , ‘The Ritual of Royal Healing in Early Modern England: Scrofula, Liturgy, and Politics’, Anglican and Episcopal History, 68, 1 (1999), 3–36 ; Simon Werrett , ‘Healing the Nation’s Wounds: Royal Ritual and Experimental Philosophy in Restoration England’, History of Science, 38, 4 (2000), 377–99. Eighteenth-century medical writers also noted the likely ‘mixture of policy’ in prior practitioners’ endorsements of the royal touch; White, *Treatise*, *op. cit.* (note 2), 3.

21

Adam Komorowski and Sang Ik Song , ‘A Politics of the Senses: The Political Role of the King’s-Evil in Richard Wiseman’s *Severall Chirurgical Treatises*
’, Medical Humanities, 45, 3 (2019), 288–9329970613 10.1136/medhum-2017-011390. See also Sturdy, ‘Royal,’ *op. cit.* (note 19), 180.

22 Komorowski and Song, ‘Politics,’ *op. cit.* (note 21), 288. The charge of plagiarism pertains to Browne’s *Compleat Treatise of the Muscles* (1681). See Ian Lyle, ‘Browne, John (1642–1702/3), Surgeon’ in *Dictionary of National Biography* online.

23 John Browne, *Adenochoiradelogia: Or, An Anatomick-Chirurgical Treatise of Glandules & Strumæs, or Kings-Evil-Swellings.* (London: Newcomb for Lowndes, 1684). Browne’s work is really a compendium of three connected treatises: the first of these, *Adenographia*, details the anatomy of the glands; the second, *Chæradelogia*, focuses on the nature and causes of ‘kings-evil-swellings’, and the third, *Charisma Basilicon*, describes ‘the royal gift of healing strumæs’. Each treatise has separate pagination; parenthetical references in this article are to the second treatise, *Chæradelogia*, unless otherwise specified.

24 Browne, *Chæradelogia, op. cit.* (note 23), 9v; see also 44.

25
*Ibid.*, 45.

26
*Ibid.*, 46.

27 For biblical uses of this phrase, see, for example, Exodus 20:5, 34:7; Numbers 14:18; Deuteronomy 5:9.

28 Browne, *Charisma Basilicon, op. cit.* (note 23), 1. On royalist attributions of scrofula to a sick body politic, see Brogan, *Royal*, *op. cit.* (note 18), 104–6; Werrett, ‘Healing the Nation’, *op. cit.* (note 20), 377–99.

29 Browne, *Chæradelogia*, *op. cit.* (note 23), 6.

30
*Ibid.*, 48, 66.

31
*Ibid.*, 48.

32
*Ibid.*, 75.

33
*Ibid.*, 44.

34
*Ibid.*, 50.

35
*Ibid.*, 9.

36

Richard Blackmore , Discourses on the Gout, a Rheumatism, and the King’s Evil (London: J. Pemberton, 1726), lx, 136.

37

Charles Peter , New Observations on the Venereal Disease (London: SD, 1695), 25 . See also, for example, John Floyer , The Ancient Psychrolousia Revived: Or, An Essay to Prove Cold Bathing Both Safe and Useful (London: Sam. Smith and Benj. Watford: 1702), 281 ; Jean Astruc, *A Treatise of the Venereal Disease*, 2 vols., trans. William Barrowby (London: W. Innys, R. Manby, *et al*., 1737), 1:138, 2:56; Richard Brookes, *The General Practice of Physic,* 2 vols (London: J. Newbery, 1754), 2:116; William Scott, *A Dissertation on the Scrofula, or Kings’-Evil* (London: M. Cooper, 1759), 8; Nicholas Rosen von Rosenstein [Nils Rosén von Rosenstein], *The Diseases of Children, and their Remedies*, trans. Andrew Sparrman (London: T. Cadell, 1776), 323.

38

Hugh Smythson , The Compleat Family Physician; or, Universal Medical Repository (London: Harrison, 1785), 29 .

39
*Ibid.*, 29.

40 On changing sexual behaviours in this period, see, for example, Tim Hitchcock, *English Sexualities, 1700-1800* (Houndmills: Macmillan, 1997); Randolph Trumbach, *Sex and the Gender Revolution* (Chicago: University of Chicago Press, 1998); Faramerz Dabhoiwala , The Origins of Sex: A History of the First Sexual Revolution (London: Penguin, 2013). On changing spending habits, see, for example, Neil McKendrick , John Brewer , and J. H. Plumb , The Birth of a Consumer Society: The Commercialization of Eighteenth-Century England (Bloomington: Indiana University Press, 1982); John Brewer and Roy Porter , Consumption and the World of Goods (London: Routledge, 1993); Michael Kwass , The Consumer Revolution, 1650–1800 (Cambridge: Cambridge University Press, 2022).

41 Browne, *Chæradelogia*, *op. cit.* (note 23), 6.

42 See, for example, Floyer, *Ancient Psychrolousia*, *op. cit.* (note 37), 280–21; Richard Blackmore, *A Treatise of Consumptions and other Distempers Belonging to the Breast and Lungs* (London: John Pemberton, 1724); Thomas Fern, *A Perfect Cure for the King’s Evil* (London: D. Browne *et al.*, 1709), 16.

43 Floyer, *Ancient Psychrolousia, op. cit.* (note 37), 281.

44 Blackmore, *Discourses on the Gout*, *op. cit.* (note 36), 136.

45

Donna Andrew , Aristocratic Vice: The Attack on Duelling, Suicide, Adultery, and Gambling in Eighteenth-Century England (New Haven: Yale University Press, 2013).

46 It is worth emphasising that medical works included case studies of wealthier and poorer sufferers, and some, like William Atkins, even suggested—contrary to popular representation—that ‘the King’s-Evil and other corrupt Diseases, are mostly among poor People.’ See William Atkins, *A Discourse Shewing the Nature of the Gout* (London: Tho. Fabian, 1694), 120. See also White, *Treatise*, *op. cit.* (note 2), 9. In the nineteenth and twentieth centuries, the disease became more closely associated with poor hygiene and thus with poor populations.

47 Jonathan Swift, *Prose Works of Jonathan Swift*, ed. Herbert Davis, 14 vols. (Oxford: Basil Blackwell, 1937–68), 2:30.

48
*Ibid.*, 2:30–1.

49
*Gulliver’s Travels* offers a notable example in describing the ‘Rottenness’ of Yahoo prostitutes who are infected with ‘a certain Malady’. See Swift, *Prose Works, op. cit.* (note 47), 11: 237.

50 For a useful overview of the critical debate on irony in the *Argument*, see Judson Curry, ‘Arguing About the Project: Approaches to Swift’s *An Argument Against Abolishing Christianity* and *A Project for the Advancement of Religion*’, *Eighteenth-Century Life,* 20 (1996), 67–79.

51 On the motif of species degeneration in *Gulliver’s Travels,* see J. Douglas Canfield, ‘Corruption and Degeneration in *Gulliver’s Travels*’, *Notre Dame English Journal,* 9, 1 (1973), 15–22; Dirk F. Passmann, ‘Degeneration in *Gulliver’s Travels*: Excavations from Brobdingnag’, *Swift Studies,* 1 (1986), 46–50; Louise Barnett , Jonathan Swift in the Company of Women (Oxford: Oxford University Press, 2006), 108–9.

52 Swift, *Prose Works, op. cit.* (note 47), 11:82–3.

53 Davidson, *Breeding*, *op. cit.* (note 14), 69–81. For an overview of the critical debate around Houhynhnm ethics, see Bernard Harrison , ‘Houhynhnm Virtue’, Partial Answers: Journal of Literature and the History of Ideas, 1, 1 (2003), 35–64 .

54 Swift, *Prose Works*, *op. cit.* (note 47), 11:240, 11:255. On genocide in Swift’s text, see Claude Rawson , God , Gulliver, and Genocide: Barbarism and the European Imagination, 1492–1945 (Oxford: Oxford University Press, 2001), esp. 256–66; John J. Burke Jr., ‘Jonathan Swift’s Crimes Against Humanity: Truth or Fiction’, 1650–1850: Ideas, Aesthetics, and Inquiries in the Early Modern Era, 10, 12 (2004), 205–16.

55 Swift, *Prose Works*, *op. cit.* (note 47), 11:240–1.

56 On marriage practices in this period, see Lawrence Stone , The Family, Sex and Marriage in England 1500–1800, abridged edn. (London: Penguin, 1990), 217–24; J. Gillis , For Better, For Worse: British Marriages 1600 to the Present Day (Oxford: Oxford University Press, 1985); R.B. Outhwaite, *Clandestine Marriage in England, 1500–1850* (London: Hambledon Press, 1985); Rebecca Probert, *Marriage Law and Practice in the Long Eighteenth Century* (Cambridge: Cambridge University Press, 2009). Stone’s argument for a shift from ‘dynastic’ to ‘companionate’ marriages has been substantially qualified by subsequent historians.

57 Ronald Paulson notes that *Marriage a-la-Mode*’s themes of arranged marriage and ill-suited partnership were already ‘commonplaces’ in the literature of the time. See Paulson, *Hogarth: His Life, Art, and Times,* 2 vols. (New Haven: Yale University Press, 1971), 1:483.

58 On the production and states of the various engravings, see Frederic George Stephens and M. Dorothy George, *Catalogue of Prints and Drawings in the British Museum*, 11 vols. (London: Trustees of the British Museum, 1870–1954), 3:2628–770.

59

Sean Shesgreen , Engravings by Hogarth (New York: Dover, 1973), 51 .

60 I focus my discussion here on the engravings, as these would have been more widely disseminated than the paintings (now housed in the National Gallery). Paulson argues that the composition of the engravings indicates the way Hogarth wanted the series to be seen: see Paulson, *Hogarth, op. cit.* (note 57), 1:478–9.

61 See N. F. Lowe , ‘The meaning of venereal disease in Hogarth’s graphic art’, in L.E. Merians (ed), The Secret Malady: Venereal Disease in Eighteenth-Century Britain and France (Lexington: The University of Kentucky, 1996), 172 .

62 See N. F. Lowe , ‘Hogarth, Beauty Spots, and Sexually Transmitted Diseases’, British Journal for Eighteenth-Century Studies, 15, 1 (1992), 71–80 ; Lowe, ‘Meaning’, *op. cit.* (note 61), 172–5; Robert L. S. Cowley , Marriage a-la-Mode: A Review of Hogarth’s Narrative Art (Manchester: Manchester University Press, 1983), 39–40 ; Noelle Gallagher, *Itch, Clap, Pox: Venereal Disease in the Eighteenth-Century Imagination* (New Haven: Yale, 2019), 36–8. For readings of the blemish as a ‘beauty spot’ or venereal sore, see, for example, Ronald Paulson, *Hogarth’s Graphic Works* (London: Print Room, 1989), 116; Judy Egerton, *Hogarth’s Marriage A-la-Mode* (London: National Gallery, 2010), 20; Shesgreen, *Engravings*, *op. cit.* (note 59), 56.

63 Stephens and George*, Catalogue, op. cit.* (note 58), 3.1:2628.

64 For readings of the sash as an anachronistic later addition, see Paulson, *Hogarth’s Graphic Works, op. cit.* (note 62), 16; Egerton, *Marriage, op. cit.* (note 62), 23; Cowley, *Marriage, op. cit.* (note 62), 42–3.

65 Critics are divided as to whether the heir is a girl (eg. Paulson) or a boy (eg. Cowley).

66 Other works influenced by *Marriage a-la-Mode* include a 1754 novel by the physician-turned-novelist John Shebbeare, *The Marriage-Act*, and David Garrick’s 1766 comedy *The Clandestine Marriage.* The 1796 print *Future Prospects*, *or Symptoms of Love in High Life* likens the ill-fated marriage between King George IV and his cousin Caroline (discussed in greater detail below) to that in Hogarth’s series.

67 Henry Fielding, Bertrand A. Goldgar, and Hugh Amory (eds.), *The Wesleyan Edition of the Works of Henry Fielding: Miscellanies,* Volume 2 (Oxford: Wesleyan, 2014), 18. On the depiction of venereal disease as ‘a la mode’, see Emily Cock, ‘The *à la mode* disease: syphilis and temporality,’ in Allan Ingram and Leigh Wetherall Dickson (eds.), Disease and Death in Eighteenth-Century Literature and Culture: Fashioning the Unfashionable (London: Palgrave, 2016), 58–9; Gallagher, *Itch, op. cit.* (note 62), 16–21.

68 Fielding, *Wesleyan*, *op. cit.* (note 67), 20.

69
*Ibid.*, 20.

70 For differing takes on how the 1753 Act worked in practice, see, for example, R.B. Outhwaite , Clandestine Marriage in England, 1500–1850 (London: Hambledon, 1995), 75–98 ; Probert, *op. cit.* (note 56), *Marriage Law.*

71 Arthur Murray, *The Gray’s Inn Journal* 70 (Saturday, February 16, 1754), 113.

72
*Ibid.*, 113–14.

73
*Ibid.*, 114.

74

Nicholas Culpeper , Culpeper’s English Physician (London: Green & Co., 1789), 169 ; William Buchan , Domestic Medicine (London: W. Strahan, 1772), 505 .

75

Charles Churchill , The Times, A Poem (London: No pub, 1764), 29 .

76

William Cowper , The Task, A Poem, in Six Books (London: J. Johnson, 1785), 166–7.

77 On the period’s anticipation of eugenics, see Hilts, ‘Enlightenment Views’, *op. cit.* (note 14), 255–71; Davidson, *Breeding, op. cit.* (note 14); Quinlan, ‘Heredity,’ *op. cit.* (note 12), 142–3; On the concept of moral panic in this period, see Christopher Hamerton, *Devilry, Deviance, and Public Sphere: The Social Discovery of Moral Panic in Eighteenth-Century London* (London: Palgrave, 2023); Quinlan, *Great Nation in Decline, op. cit.* (note 12).

78 It is worth noting that Charles II and James II were also accused of contracting or spreading venereal disease. See Leon Guilhamet, ‘Pox and Malice: Some Representations of Venereal Disease in Eighteenth-Century Satire’, in L.E. Merians (ed), *The Secret Malady: Venereal Disease in Eighteenth-Century Britain and France* (Lexington: The University of Kentucky, 1996), 203–5; Gallagher, *Itch*, *op. cit.* (note 62), 74–6.

79 Gallagher, *Itch*, *op. cit.* (note 62), 81–92.

80 Isaac Swainson, *Directions for the Use of Velnos’ Vegetable Syrup* (London: no pub, 1787), 2, 11, 14, 23. For images of George with Velno’s, see, for example, James Gillray, *A Voluptuary Under the Horrors of Digestion* (1792), *A New Bravura with a Duett Affettuoso* (1802), both in the British Museum.

81 On this image, see Stephens and George, *Catalogue*, *op. cit.* (note 58), 7:8610.

82

Claude Quillet , Callipædia, Or , The Art of Getting Pretty Children (London: Bernard Lintot, 1710), 9.

83
*Ibid.*, 9. The 1720 edition includes epilepsy, cholic, and ulcers, as well as gout and consumption, as conditions that render a person unfit for childbearing; see Claude Quillet , Callipædia (London: Bernard Lintot, 1720), 22 .

84 Quillet, *Callipædia*, 11.

